# Supplementary material for: Transthoracic ultrasound localization microscopy of myocardial vasculature in patients
Source: Nat Biomed Eng. 2024 May 6;8(6):689–700. doi: 10.1038/s41551-024-01206-6 (PMC11250254; doi:10.1038/s41551-024-01206-6)
Supplement: Supplementary file 1 — Supplementary methods, figures, tables and video captions. [file 41551_2024_1206_MOESM1_ESM.pdf]

nature biomedical engineering

Article

https://doi.org/10.1038/s41551-024-01206-6

Transthoracic ultrasound localization microscopy of myocardial vasculature in patients

In the format provided by the authors and unedited

Contents

Supplementary Methods
Supplementary Figs. 1–5
Supplementary Tables 1 and 2
Captions for the supplementary videos

Supplementary Methods: Fuzzy Initialisation and Parameter Estimation for Tracking based on Kalman Motion Model

In this supplement, we explain the impacts of microbubble (MB) concentrations and ultrasound acquisition frame rates on MB tracking, giving the reasons for developing the methods and unifying concentrations and frame rates as one equivalent factor in tracking problem; present the method for Kalman state vector initialisation; and present the method for Kalman parameter estimation.

1. Shannon theory in MB tracking

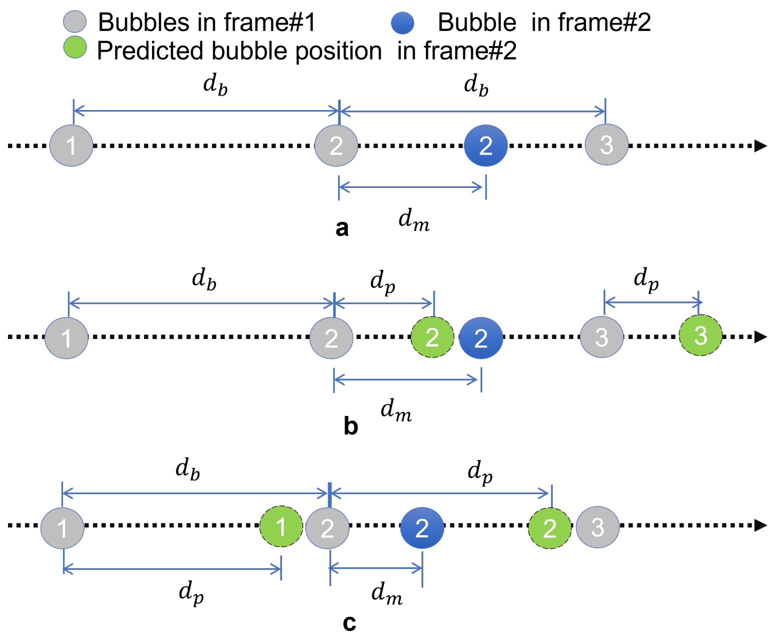

Fig. M1 | ** Three cases to pair multiple MBs moving in one straight vessel. **a, a case where MBs are to be paired without prediction of motion. **b,** a case where MBs are to be paired by motion model with predicted movement less than the moving distance. **c,** a case where MBs are to be paired by motion model with predicted movement more than the moving distance. Number in each disk denotes the identity of an MB. d_b is the distance between MBs in one frame. d_m is the moving distance of the MB between two frames. d_p is the moving distance predicted by motion model for MBs.

The relation between Shannon theory and the tracking can be revealed by simple cases, where multiple MBs move in a straight vessel, as shown in Fig. M1a. To correctly pair MB 2 by the nearest-neighbour method, the distance between the positions of MB 2 in two frames should be smaller than the distance between MB 2 in the second frame and MB 3 in the first frame.

$$d_m < d_b - d_m \quad i.e. \quad d_m < 0.5d_b \tag{1}$$

where d_m is the moving distance, the ratio of flow speed to frame rate, and d_b is the MB distance which is inverse to the MB concentration. According to the above equation, a higher frame rate is needed to track MBs at higher concentrations and faster speeds. Motion model can predict the MB position along the flow direction, as shown in Fig. M1b. To correctly pair MB 2 in this case, the distance between the MB 2's predicted position and actual positions should be smaller than the distance between the MB 3's predicted position and MB 2's actual position,

$$d_m - d_p < d_b + d_p - d_m \quad i.e. \quad d_m < 0.5d_b + d_p \tag{2}$$

where d_p is the MB moving distance predicted by a motion model. With the motion model, tracking method can correctly pair faster MBs at the same frame rate and MB concentrations, compared to the nearest-neighbour method. However, it doesn't mean the larger the d_p is, the higher accuracy the tracking can achieve. To avoid pairing MB 2 in Frame 2 to MB 1 in Frame 1 in the case shown in Fig. M1c, d_p should satisfy

$$d_p - d_m < d_b - d_p + d_m \quad i.e. \quad d_p < 0.5d_b + d_m \tag{3}$$

Combining Eq. (2) and (3), we can obtain

$$|d_m - d_p| < 0.5d_b \tag{4}$$

With the frame rate *f*, the equation can be rewritten as

$$\frac{2|v_m - v_p|}{d_b} < f \tag{5}$$

where v_m is the flow velocity and v_p is the predicted velocity and v_m/d_b can be regarded as the spatial repetition frequency, defined by how many times an MB passes through the positions of the other MBs in one second. Then, the temporal frequency, frame rate or pulse repetition frequency *f*, should be at least double the spatial repetition frequency to correctly pair the MB by nearest-neighbour. Using the motion model, the spatial repetition frequency of the MB movement v_m/d_b can be reduced by v_p/d_b.

Fast-moving MBs at high concentrations can be more accurately tracked using motion model than only using the nearest-neighbour, especially when the frame rates are restricted by physical imaging depth and the number of angles used for compounding.

2. Fuzzy initialization

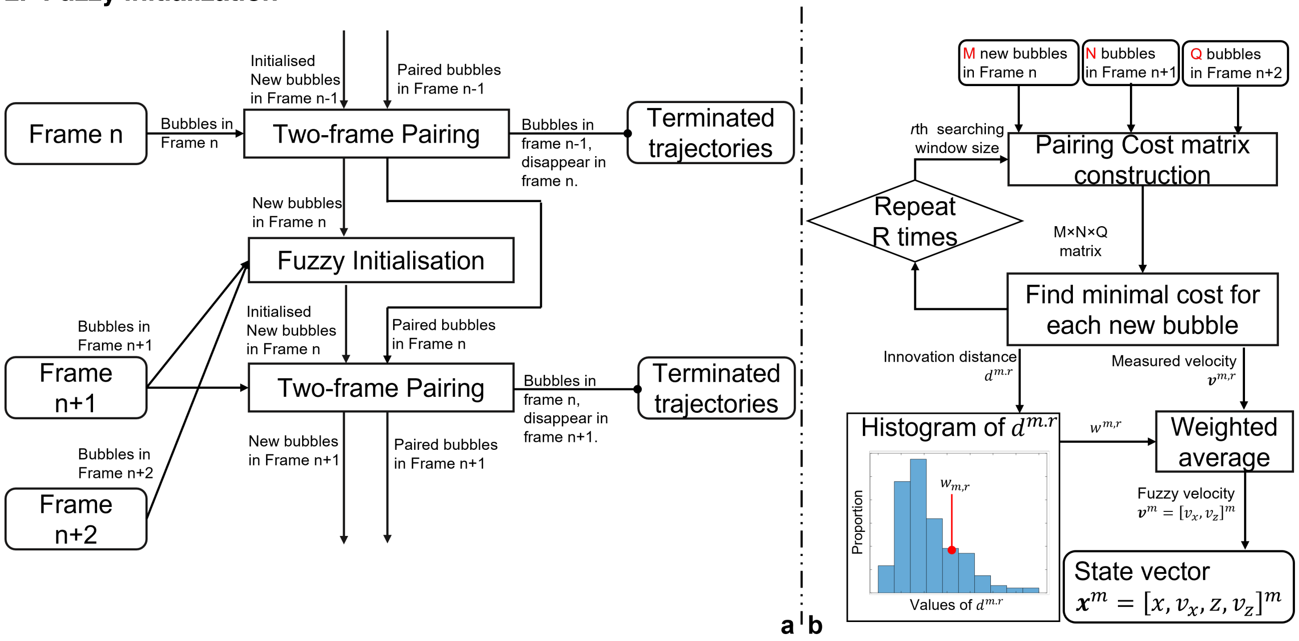

Fig. M2 I a, a framework combining fuzzy initialisation and graph-based assignment for MB tracking. Fuzzy initialisation is done for new MBs detected in frame n by using MBs in frame n, n+1 and n+2. After initialisation, all MBs in frame n are paired with MBs in frame n+1 by our previously proposed feature-motion-model tracking framework. **b,** fuzzy initialisation diagram. Moving velocities of a newly appeared MB is estimated with MBs in next two consecutive frames by finding minimum of pairing cost under different searching window sizes. Weighted average of the velocities obtained under different searching window sizes is used as the velocity components in the Kalman state vector of the new MB. $v^{m,r}$ is measured velocity between the first two frames corresponding to the minimal cost found for the *m*th new MB under the *r*th searching window size. Innovation distance $d^{m,r}$ in the third frame corresponds to the minimal cost found for the *m*th new MB under the *r*th searching window size.

In our previous work [1], we proposed a feature-motion-model framework to achieve accurate and efficient MB tracking, which combined MB image feature and Kalman motion model in a cost function and paired MBs by finding the total minimum cost in a graph-based assignment. The Kalman motion model's state vector **x**, translation matrix **F** and observation matrix **H** is defined as

$$\mathbf{F} = \begin{bmatrix} 1 & \frac{1}{f} & 0 & 0 \\ 0 & 1 & 0 & 0 \\ 0 & 0 & 1 & \frac{1}{f} \\ 0 & 0 & 0 & 1 \end{bmatrix}, \quad \mathbf{x} = \begin{bmatrix} x \\ v_x \\ z \\ v_z \end{bmatrix}, \quad \mathbf{H} = \begin{bmatrix} 1 & 0 & 0 & 0 \\ 0 & 0 & 1 & 0 \end{bmatrix} \tag{6}$$

where x and z are the lateral and depth location of MBs in the image; v_x and v_z are the MB moving speeds along the corresponding directions. v_x and v_z, i.e. v_p, were set to 0 for the newly appeared MBs as we could not get the MB velocity before pairing. As demonstrated in the last section, wrong pairing is still likely to happen for new MB with this initialisation where motion model does not take effect by assuming new MB to be static. Therefore, we proposed fuzzy initialisation and combined it with our previous framework in the way shown in Fig. M2a. The fuzzy initialisation was done for the newly appeared MBs using three consecutive frames, as shown in Fig. M2b. The fuzzy initialization was done with the below steps.

Cost matrix construction. To initialise the M new MBs in the first frame, a 3D cost matrix for paring the M new MBs with the MBs in the following two frames was built. Various information can be used for calculating the cost. In this study, the cost consisted of three components: innovation of motion model, intensity differences, and intensity variances along the candidate tracks.

We estimated v_x and v_z for each new MB in the first two frames, and therefore the innovation of motion model could be calculated by the distance between the observed MB locations and the predicted MB locations in third frame

$$d^{m,n,q} = \sqrt{(x^q + x^m - 2x^n)^2 + (z^q + z^m - 2z^n)^2} \tag{7}$$

where the superscripts, $m \leq M$, $n \leq N$ and $q \leq Q$, denote the MB indexes in the three frames. When giving a search window, model innovations for pairs out of the searching window were given as infinity.

The intensity difference d_I among MBs in three frames was calculated as

$$d_I^{m,n,q} = |I^q + I^m - 2I^n| \tag{8}$$

where I is the MB image intensity.
The maximum intensity projection (MIP) of contrast enhanced ultrasound (CEUS) sequence presents the vasculature in a low resolution. While pairing two MBs, the trajectory can be along or across two vessels. It can be assumed that pixel intensities on the trajectory along one vessel have smaller variance than those on trajectories across vessels. Therefore, the intensity variances of MIP pixels on each trajectory can be used for calculating the cost of pairing and defined as

$$\sigma_p^{m,n,q} = \frac{\sigma_{pt}}{\mu_{pt}} \tag{9}$$

where the numerator is the standard deviation of pixel intensities on a trajectory and the denominator is the average. To save computation, the trajectory between one pair was defined as a straight line; seven points were equidistantly sampled on the trajectory between MB pairs in the first two frames and then assigned to candidate pairs among three frames which shared the same pairing between the first two frames; the pixel intensity of the points on each trajectory was obtained from the nearest MIP pixel.
Three components were in the same size of 3D matrix and combined to a 3D matrix by the first-order principal components of the principal components analysis (PCA).

Finding the minimum. The minimal cost was found for each new MB independently, instead of finding the total minimum of all the pairs to save the computation. The innovation distance d^m and velocity vector v^m between MBs in the first two frames corresponding to the minimal cost were saved for each new MB.

Repetition within different sizes of searching windows. To increase the robustness of the initial paring, the above two steps were repeated for different search window sizes. In this study, the searching window sizes were varied between 0.3 to 1.2 times the maximum MB moving distance between adjacent frames (assuming maximal blood flow velocity of 150 mm/s). As a result, for the rth searching window, $d^{m,r}$ and $\mathbf{v}^{m,r}$ were saved for the mth new MB.

Initialising Kalman state vector. An intuitive way to combine multiple $\boldsymbol{v}^{m,r}$ into one for the new MB is averaging. However, the distribution of $\boldsymbol{v}^{m,r}$ may not be Gaussian, and some extreme cases might significantly affect the average. As the distance $d^{m,r}$ can be regarded as errors of the linear motion model, the normalised histogram, shown in Fig. M2b, represents the probability of the certain value of errors when assuming linear MB movements for this data. Therefore, the velocity of the new MB can be calculated by the below weighted average to reduce the effect of extreme cases

$$\boldsymbol{v}^m = \frac{\sum_{r=1}^{R} w^{m,r} \boldsymbol{v}^{m,r}}{\sum_{r=1}^{R} w^{m,r}} \tag{10}$$

where $w^{m,r}$ is the number obtained by indexing the histogram by $d^{m,r}$ corresponding to the $\boldsymbol{v}^m$, and R is the number of searching window sizes used. A new bubble might not be paired with any other bubble in a small searching window. In this case, the searching window was excluded from the average in Eq. (10). Only the new bubbles were paired in more than two searching windows were initialised by Eq. (10), and otherwise were set as static.

In the above initialisation, the speed components in the state vector were obtained by the weighted average across the different searching window sizes. As a result, a velocity vector from an initialised MB might not point to any MB in the next frame. Therefore, we named the above procedures as fuzzy initialisation.

After being initialised, new MBs in current frame and MBs existing in previous as well as current frames were paired with all the MBs in the next frame by finding the total minimum of cost through the graph-based assignment. The cost for the graph-based assignment was defined as the ratio of MB image intensity difference to the probability obtained through motion model by $p(o_k|x_{k|k-1})$

$$p(\boldsymbol{o}_k|\boldsymbol{x}_{k|k-1}) = \frac{1}{2\pi|\mathbf{S}_k|^{0.5}} \exp\left(-0.5(\boldsymbol{o}_k - \boldsymbol{\mu})^{\mathrm{T}} \mathbf{S}_k^{-1} (\boldsymbol{o}_k - \boldsymbol{\mu})\right)$$
$$\boldsymbol{\mu} = \mathbf{H}\hat{\boldsymbol{x}}_{k|k-1} \tag{11}$$

where the subscript $n|n'$ denotes the estimate of the value in frame n given observations up to and including frame n'; o_k is measured MB state vector, consisting of lateral and depth coordinates; $\mathbf{S}_k$ is the innovation covariance.

3. Kalman motion model parameters

Covariance of measurement noise **R**, covariance of model prediction noise **Q** and covariance of estimation noise P are parameters that need to be set for the linear Kalman motion model and sometimes can significantly reduce tracking accuracy with inappropriate values. **R** can be set by the localization uncertainty of the imaging system, which can be measured by *in vitro* experiment and estimated for *in vivo* acquisition. Although **Q** can be manually chosen according to user experiences, we proposed a method to estimate **Q** from data to avoid human interaction and reduce effort in tunning parameters when dealing with various vasculature and haemodynamics.

As presented in the last section, a linear motion model is used to predict MB movement in the third frame using the first two frames. Steps from 1 to 3 in the last section can be used for to estimate **Q** for each three adjacent frames. Instead of saving distance between the prediction and observation for each MB, the discrepancies along lateral $e^{x,m} = (x^q + x^m - 2x^n)$ and depth $e^{z,m} = (z^q + z^m - 2z^n)$ were saved for each MB in the first of the three frames. By assuming most of pairing after these three steps are correct, the discrepancies can be used to approximate the measurement pre-fit residual of Kalman filtering, the distance between predicted and measured MB locations. Therefore, we used the variance of the discrepancies to calculate the pre-fit residual covariance.

$$\hat{\mathbf{S}} = \begin{bmatrix} \sigma_{ex}^2 & 0 \\ 0 & \sigma_{ez}^2 \end{bmatrix} \tag{12}$$

where σ_{ex}^2 and σ_{ez}^2 are the variance across $e^{x,m}$ and $e^{z,m}$ respectively. As variance estimation becomes more accurate with a larger number of data, discrepancies were obtained from ten groups of three consecutive frames picked from the image sequence, and a variance σ_e^2 was calculated with all the discrepancies along the lateral and axial directions and used for σ_{ex}^2 and σ_{ez}^2.

The **S** in one frame was defined by

$$\mathbf{S}_k = \mathbf{H}\mathbf{P}_{k|k-1}\mathbf{H}^{\mathrm{T}} + \mathbf{R}$$
$$\mathbf{P}_{k|k-1} = \mathbf{F}\mathbf{P}_{k-1|k-1}\mathbf{F}^{\mathrm{T}} + \mathbf{Q} \tag{13}$$

where $\mathbf{P}_{k-1|k-1}$ and $\mathbf{P}_{k|k-1}$ are the updated and predicted estimation covariance matrices respectively. Using optimal Kalman gain, $\mathbf{P}_{k-1|k-1}$ is independent from the measurements and can achieve an asymptotic value as

$$\mathbf{P}_{\infty} = \mathbf{F}(\mathbf{P}_{\infty} - \mathbf{P}_{\infty}\mathbf{H}^{\mathrm{T}}(\mathbf{H}\mathbf{P}_{\infty}\mathbf{H}^{\mathrm{T}} + \mathbf{R})^{-1}\mathbf{H}\mathbf{P}_{\infty})\mathbf{F}^{\mathrm{T}} + \mathbf{Q} \tag{14}$$

Therefore, $\mathbf{P}_{\infty}$ is determined by the defined **R** and **Q** and used for $\mathbf{P}_{k-1|k-1}$ to make MB Kalman filter independent from how many frames the MB have appeared in the imaging plane. The time continuous noise model [2] was used and thus

$$\mathbf{Q} = \begin{bmatrix} \frac{1}{3f^3} & \frac{1}{2f^2} & 0 & 0 \\ \frac{1}{2f^2} & \frac{1}{f} & 0 & 0 \\ 0 & 0 & \frac{1}{3f^3} & \frac{1}{2f^2} \\ 0 & 0 & \frac{1}{2f^2} & \frac{1}{f} \end{bmatrix} q^2 \tag{15}$$

where q is the variance of error when assuming constant velocity in the linear Kalman motion model. As analytical solution of Eq. (14) was not found, **Q** was estimated using Eq. (15) while q was calculated by the bisection method in Algorithm 1.

Algorithm 1 Find q

Require: **R**, $|\hat{S}|$, $[q_b, q_u]$ searching range of q
  while $q_u - q_b > \epsilon$ **do**
    $q \leftarrow (q_b + q_u)/2$
    Calculate $\mathbf{P}_{\infty}$ with **Q** and **R**
    Calculate **S** with Eq. (13)
    if $|S| > |\hat{S}|$ **then**   ➢ $|S|$ increases with q in the searching range
      $q_u \leftarrow q$
    else
      $q_b \leftarrow q$
    end if
  end while
Return q

References

[1] J. Yan, T. Zhang, J. Broughton-Venner, P. Huang, and M.-X. Tang, "Super-resolution ultrasound through sparsity-based deconvolution and multi-feature tracking," *IEEE Transactions on Medical Imaging*, vol. 41, no. 8, pp. 1938–1947, 2022.

[2] D. Ackermann and G. Schmitz, "Detection and tracking of multiple microbubbles in ultrasound b-mode images," *IEEE transactions on ultrasonics, ferroelectrics, and frequency control*, vol. 63, no. 1, pp. 72–82, 2015.

Supplementary figures

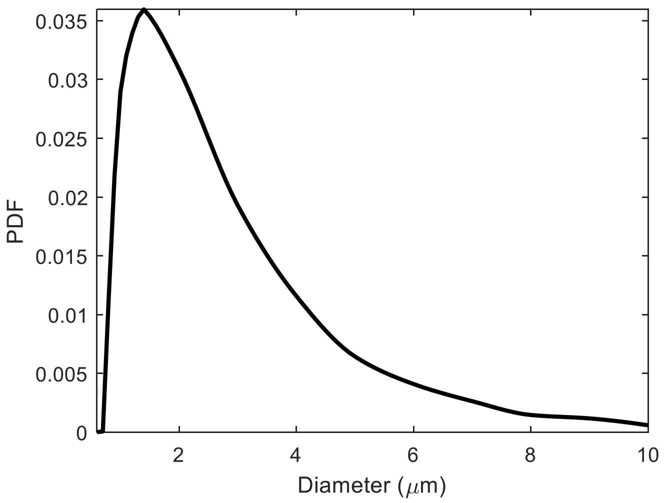

Supplementary Fig. 1 | Diameter distribution of Sonovue microbubbles. PDF: probability density function

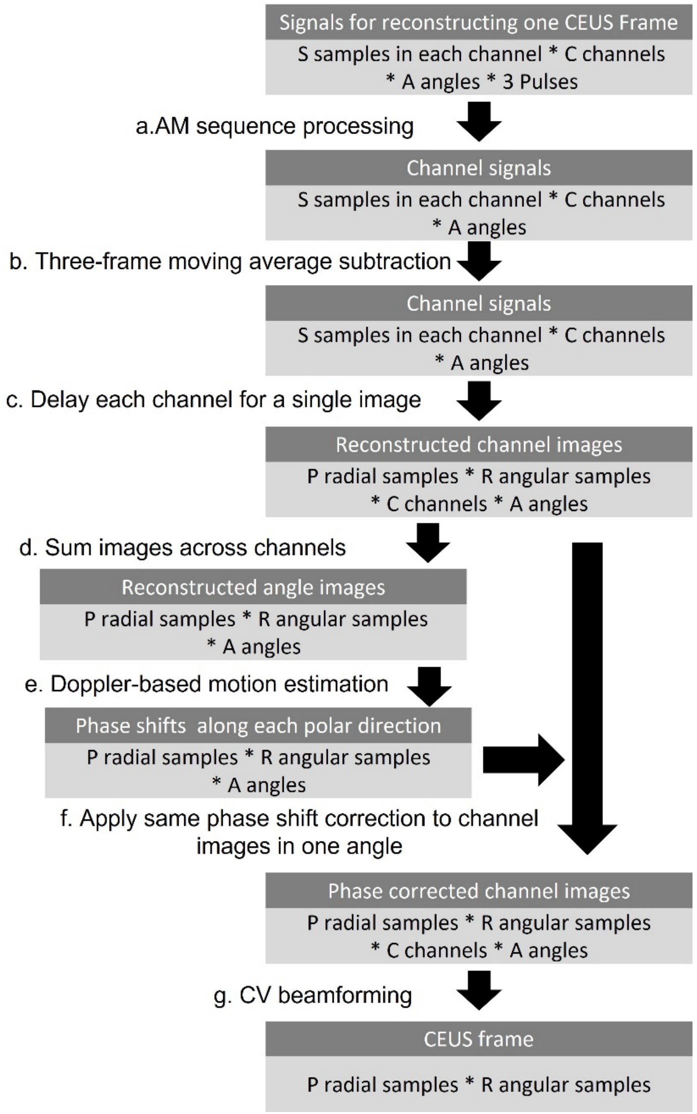

Supplementary Fig. 2 l CEUS image generation and processing. **a**, AM was done to remove linear tissue signals. **b**, moving average subtraction was done to remove remaining nonlinear tissue signals. **c**, image in polar coordinate was generated for each angle and each channel. **d**, images were summed across channels corresponding to each angle. **e**, MB shifts during steering angles were estimated by the Doppler-based method. **f**, MB motions in each channel images were corrected by the phase shifts estimated from the corresponding steering angles. **g**, CEUS images were reconstructed with the coherence to variance (CV) beamforming, where the variance was calculated across channels and steering angles for each pixel.

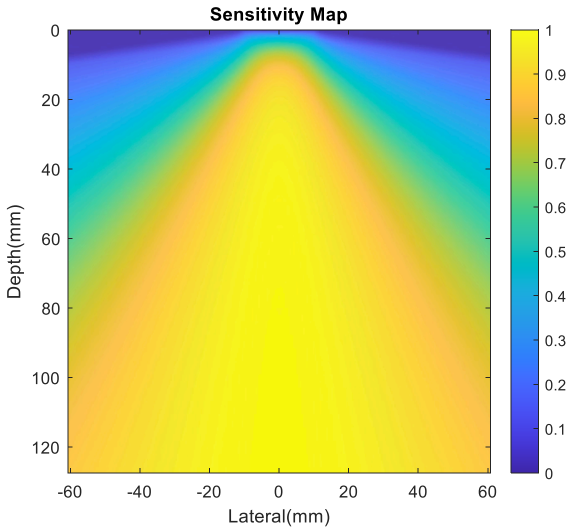

Supplementary Fig. 3 l Probe sensitivity map. The map was obtained by summing all the element directivities to each pixel. The element sensitivity can be described as $sinc\left(\frac{L}{\lambda}sin(\theta)\right) \times cos(\theta)$, where L is the element width; λ is the wavelength; θ is the angle between the vertical direction and the line connecting the element centre and pixel.

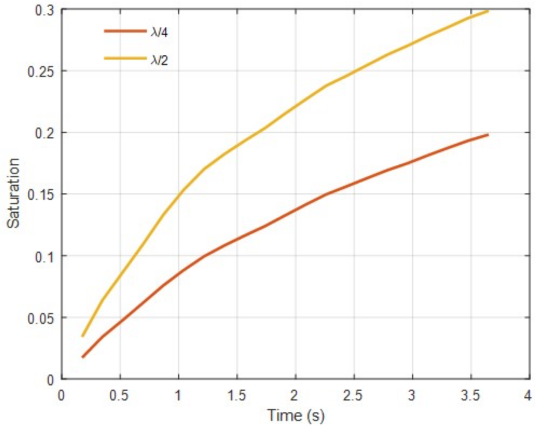

Supplementary Fig. 4 l Saturation curve of the short-axis data of Patient 1 versus the acquisition time. Maps with pixel size of half or a quarter wavelength (λ) were filled by localizations. Saturation was calculated by the ratio of filled area to all the area in the myocardium. The figure demonstrates that the curves do not reach a plateau and the SR images are not fully saturated.

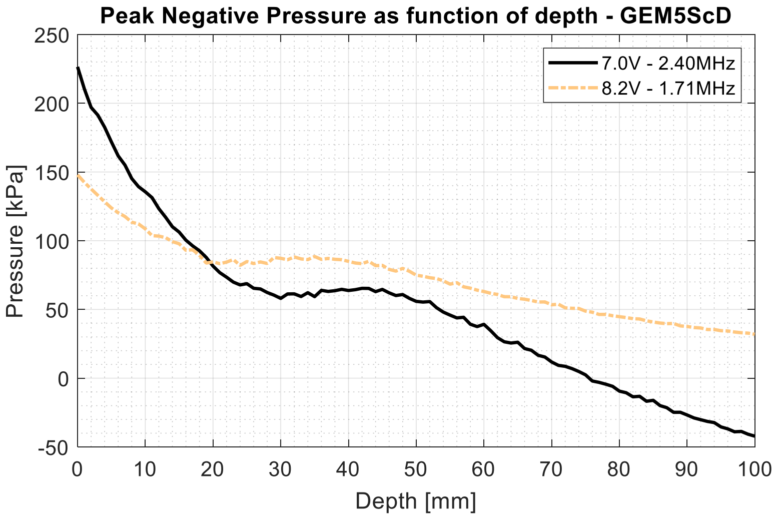

Supplementary Fig. 5 | Pressure measured at different depths in a water tank along the central line of the GEM5 ScD probe. The ultrasound pulses centred at 2.4 MHz was transmitted at 7 V, and the pulses centred at 1.7 MHz was transmitted at 8.2 V when acquiring data. Pressure was measured by a needle hydrophone (0.2 mm, Precision Acoustic, UK). The measured pressure was derated by 0.3 dB/MHz/cm to get the curves in the figure, considering acoustic attenuation in tissues.

Supplementary tables

Supplementary Table 1 I Parameters and components used for acquisition and processing. More details can be found in Methods and Supplementary Methods.

		Parameter/ Component	Value/ Brief Description
Acquisition with GEM5ScD probe		Transmitted entre frequency	2.4 MHz or 1.7 MHz
		Diverging wave focus	-21.6 mm
		Steering angle sequence	-15°, -3°, 9°, 15°, 3°, -9°
		Driving voltage	7 V or 8.2 V, measured pressures are shown in Fig. F10
		AM sequence (Half-Full-Half aperture)	Interleaved groups with two elements, i.e., driving elements as (11001100…) or (00110011…) for half amplitude.
		Frame rate	305 Hz
		Duration	10 second
Multi-level Motion Correction	CEUS reconstruction with MB MoCo (Diagram shown in Fig. F5)	Polar grid	67.8 μm and 0.5°
		Doppler window size	1 mm and 3.5°
	Intra-cycle tissue MoCo (two-stage registration: affine + B-spline-based non-rigid)	Image difference metric	Sum of square pixel intensity difference
		MB signal reduction	Keep 5% largest singular values of SVD for reconstruction
		B-mode image dynamic range	50 dB after log compression
		Cartesian grid	Depth: 67.8 μm. Lateral: 135.0 μm
		B-spline grid	Depth: 2.17 mm. Lateral: 4.32 mm
		Solver	Affine: Levenberg–Marquardt algorithm Non-rigid: Steepest decent algorithm
		Maximum iteration steps	500
		Stopping criteria	Image difference between two adjacent iterations changed less than 0.001% for 20 times.
	Inter-cycle tissue MoCo (Rigid registration)	Averaged CEUS dynamic range	100 dB after log compression
		The other parameters were same with the affine registration used for the intra-cycle motion correction	
Localization		Cartesian grid	13.5 μm in both directions
		Threshold on normalised cross-correlation coefficient map	0.5
Tracking		Searching window size	150 mm/s
		Kalman parameters	Estimated from the data. Details can be found in the supplementary method.
		MB persistence filtering	>3 frames

Supplementary Table 2 I Localization uncertainty measurements with in vitro experiments. A wire target was fixed under three different distances from the probe and imaged for 600 frames per depth using the same acquisition settings of the in vivo experiments. Localization uncertainties were quantified by the variance of localised positions at each depth. Both transmission frequencies, i.e., 1.7 and 2.4 MHz, used in in vivo acquisition were tested.

Depth (mm)	1.7 MHz		2.4 MHz	
	Lateral uncertainty (um)	Axial uncertainty (um)	Lateral uncertainty (um)	Axial uncertainty (um)
30	1.94	1.23	7.66	9.09
80	4.80	2.48	3.29	3.02
120	9.40	1.84	15.54	1.17

Captions for the Supplementary Videos

Supplementary Video 1 l Short-axis view B-mode frames of Porcine 1 before (left) and after (right) motion correction. Dynamic range: 50 dB.

Supplementary Video 2 l Short-axis view B-mode frames in one diastole phase of Patient 1 before (left) and after (right) motion correction. Dynamic range: 50 dB.

Supplementary Video 3 l Long-axis view B-mode frames in one diastole phase of Patient 1 before (left) and after (right) motion correction. Dynamic range: 50 dB.

Supplementary Video 4 l Demonstration of localization and tracking on short-axis view CEUS frames of Porcine 1. Red lines denote trajectories of microbubbles detected on current frames. Dynamic range of CEUS images: 100 dB.

Supplementary Video 5 l Demonstration of localization and tracking at the top-left region on short-axis view CEUS frames of Patient 1. Right: red crosses denote localised positions of MBs. Left: Flow speed map with temporally accumulated MB trajectories after persistence filtering. Dynamic range of CEUS images: 100 dB

Supplementary video 6 l Image/video sequence in Supplementary video 6 in the sequence of appearance: 1) Acquisition position demonstration. 2) B-mode frames of two consecutive cardiac cycles. 3) CEUS frames of the two consecutive full cardiac cycles obtained with moving-average subtraction and DAS beamforming. 4) CEUS frames in four consecutive diastoles obtained with angle MoCo and CV beamforming. 5) SR density maps. 6) Comparison with CTCA. 7) Microcirculation visualisation. Microcirculation visualisation was done by moving localised microbubbles along their trajectories. Each scatter in the visualisation represents one localised microbubble.
