# Supplementary material for: Transthoracic ultrasound localization microscopy of myocardial vasculature in patients
Source: Nat Biomed Eng. 2024 May 6;8(6):689–700. doi: 10.1038/s41551-024-01206-6 (PMC11250254; doi:10.1038/s41551-024-01206-6)
Supplement: Supplementary file 2 — Reporting Summary [file 41551_2024_1206_MOESM2_ESM.pdf]

nature portfolio

Corresponding author(s): Meng-Xing Tang

Last updated by author(s): Mar 8, 2024

Reporting Summary

Nature Portfolio wishes to improve the reproducibility of the work that we publish. This form provides structure for consistency and transparency in reporting. For further information on Nature Portfolio policies, see our Editorial Policies and the Editorial Policy Checklist.

Statistics

For all statistical analyses, confirm that the following items are present in the figure legend, table legend, main text, or Methods section.

n/a	Confirmed	
☐	☒	The exact sample size (*n*) for each experimental group/condition, given as a discrete number and unit of measurement
☐	☒	A statement on whether measurements were taken from distinct samples or whether the same sample was measured repeatedly
☐	☒	The statistical test(s) used AND whether they are one- or two-sided<br>*Only common tests should be described solely by name; describe more complex techniques in the Methods section.*
☒	☐	A description of all covariates tested
☒	☐	A description of any assumptions or corrections, such as tests of normality and adjustment for multiple comparisons
☐	☒	A full description of the statistical parameters including central tendency (e.g. means) or other basic estimates (e.g. regression coefficient) AND variation (e.g. standard deviation) or associated estimates of uncertainty (e.g. confidence intervals)
☐	☒	For null hypothesis testing, the test statistic (e.g. *F*, *t*, *r*) with confidence intervals, effect sizes, degrees of freedom and *P* value noted<br>*Give P values as exact values whenever suitable.*
☒	☐	For Bayesian analysis, information on the choice of priors and Markov chain Monte Carlo settings
☒	☐	For hierarchical and complex designs, identification of the appropriate level for tests and full reporting of outcomes
☒	☐	Estimates of effect sizes (e.g. Cohen's *d*, Pearson's *r*), indicating how they were calculated

Our web collection on statistics for biologists contains articles on many of the points above.

Software and code

Policy information about availability of computer code

Data collection	Custom data-acquisition codes developed in Matlab2022a are available on reasonable request.
Data analysis	Code for the Doppler-based motion correction method was proposed in J. Porée, D. Posada, A. Hodzic, F. Tournoux, G. Cloutier and D. Garcia, "High-Frame-Rate Echocardiography Using Coherent Compounding With Doppler-Based Motion-Compensation," in IEEE Transactions on Medical Imaging, vol. 35, no. 7, pp. 1647-1657, July 2016.<br><br>We used existing B-spline free-form registration. A CPU code version can be found in Matlab Central File Exchange (https://www.mathworks.com/matlabcentral/fileexchange/20057-b-spline-grid-image-and-point-based-registration). We developed a CUDA version (V11.4.120) for faster computation.<br><br>Executable super-resolution software for general super-resolution processing containing all key SRUS processing steps, as well as a graphical user interface, can be found on GitHub at https://github.com/JipengYan1995/SRUSSoftware. The software consists of data loading, motion correction, super-localization, tracking, plotting, parameter calculation, and animation generation. Detailed descriptions and user guides of the software can be found in the repository.

For manuscripts utilizing custom algorithms or software that are central to the research but not yet described in published literature, software must be made available to editors and reviewers. We strongly encourage code deposition in a community repository (e.g. GitHub). See the Nature Portfolio guidelines for submitting code & software for further information.

Data

Policy information about availability of data

All manuscripts must include a data availability statement. This statement should provide the following information, where applicable:

- Accession codes, unique identifiers, or web links for publicly available datasets
- A description of any restrictions on data availability
- For clinical datasets or third party data, please ensure that the statement adheres to our policy

A sample-image dataset and step-and-step instructions for using the data are available from figshare at https://doi.org/10.6084/m9.figshare.22331635. Raw data samples are available from the SRUSSoftware Github repository at https://github.com/JipengYan1995/SRUSSoftware.

Research involving human participants, their data, or biological material

Policy information about studies with human participants or human data. See also policy information about sex, gender (identity/presentation), and sexual orientation and race, ethnicity and racism.

Reporting on sex and gender	Sex and gender were not considered in the study design.
Reporting on race, ethnicity, or other socially relevant groupings	Socially relevant variables were not considered in the study design.
Population characteristics	Four patients were recruited from the cardiac outpatient clinic after giving informed and written consent to participate in a prospective clinical cohort study investigating novel methods for non-invasive arrhythmogenic substrate characterization including high-frame-rate contrast-enhanced transthoracic echocardiography.<br><br>Patient 1 was a 60-year-old male (BMI 24 kg/m2 – 167 cm, 67 kg) with typical hypertrophic cardiomyopathy and with a secondary prophylactic left-sided dual chamber implantable cardioverter defibrillator (ICD). (Fig. 3, Fig. 5b).<br><br>Patient 2 was a 31-year-old male (BMI 23.3 kg/m2 – 191 cm, 83.0 kg) with dilated left ventricular cavity (139.57ml/m2, but preserved left ventricular ejection fraction (LVEF): 63%), no regional wall motion abnormalities, without evidence of myocardial scar in the cardiac MRI. The left ventricular dilatation was likely secondary to high burden of ventricular ectopy of > 40%. (Fig. 4).<br><br>Patient 3 was a 31-year-old male (BMI 25.3 kg/m2 – 175 cm, 77.6 kg) with background of idiopathic ventricular fibrillation with structurally normal heart in cardiac MRI and secondary prevention transvenous dual chamber ICD insertion who presented with appropriate ICD shocks due to recurrent ventricular ectopic triggered ventricular fibrillation. (Extended Data Fig.4a,b,c).<br><br>Patient 4 was a 34-year-old male (BMI 28.9 kg/m2 - 178.5cm, 72.8kg) with hypertrophic cardiomyopathy with asymmetric septal hypertrophy (max. wall thickness 21mm) without outflow tract obstruction, LVEF 75%, and a with a primary prevention subcutaneous ICD. Cardiac MRI reported patchy LGE throughout the hypertrophied septum as well as large dense perfusion defects throughout the septum during a perfusion study with adenosine. (Extended Data Fig.4e,f,g)
Recruitment	Patients were recruited from the cardiac outpatient clinic after giving informed and written consent to participate in a prospective clinical cohort study investigating novel methods for non-invasive arrhythmogenic substrate characterization including high-frame-rate contrast-enhanced transthoracic echocardiography. Criteria to select the patients in this study include a good acoustic window and signal-to-noise ratio in the CEUS data judged visually by the operator. Such selection shouldn't impact the conclusions of the study, as this is a feasibility study (with no statistics comparing patient groups, for example).
Ethics oversight	The study was reviewed and approved by the London-Bromley Research Ethics Committee and the Health Research Authority (IRAS Project ID 144257, REC reference 14/LO/0360) and is ongoing.

Note that full information on the approval of the study protocol must also be provided in the manuscript.

Field-specific reporting

Please select the one below that is the best fit for your research. If you are not sure, read the appropriate sections before making your selection.

☒ Life sciences ☐ Behavioural & social sciences ☐ Ecological, evolutionary & environmental sciences

For a reference copy of the document with all sections, see nature.com/documents/nr-reporting-summary-flat.pdf

Life sciences study design

All studies must disclose on these points even when the disclosure is negative.

Sample size	No sample-size calculation was performed as this study does not present any statistical analysis between patients. The study shows the feasibility of transthoracic super-resolution ultrasound localization microscopy in all the patients (N=4) with comparison to available CTCA or cardiac MRI. A sample size of 4 is appropriate for proof of concept.
Data exclusions	No data were excluded.
Replication	For the proof-of-concept experiments, myocardial vasculature was successfully reconstructed from four patients. The ultrasound probe was held by an experienced clinician to target the imaging plane. The starting time of acquisition needed to be delayed after microbubble injection to await for the microbubbles to perfuse the myocaridum. Codes and software were validated by simulations, phantoms and ex vivo experiments before clinical experiments.
Randomization	Randomization was not relevant to the study, as it only aimed to show the feasibility of transthoracic super-resolution ultrasound localization microscopy in patients. All the patients were allocated in a single group, and no statistical analyses between patients were carried out.
Blinding	Blinding was not relevant to the study.

Reporting for specific materials, systems and methods

We require information from authors about some types of materials, experimental systems and methods used in many studies. Here, indicate whether each material, system or method listed is relevant to your study. If you are not sure if a list item applies to your research, read the appropriate section before selecting a response.

Materials & experimental systems

n/a	Involved in the study
☒	☐ Antibodies
☒	☐ Eukaryotic cell lines
☒	☐ Palaeontology and archaeology
☐	☒ Animals and other organisms
☒	☐ Clinical data
☒	☐ Dual use research of concern
☒	☐ Plants

Methods

n/a	Involved in the study
☒	☐ ChIP-seq
☒	☐ Flow cytometry
☒	☐ MRI-based neuroimaging

Animals and other research organisms

Policy information about studies involving animals; ARRIVE guidelines recommended for reporting animal research, and Sex and Gender in Research

Laboratory animals	Two porcine hearts were explanted from large white pigs (65–75kg, 4–5-months old).
Wild animals	The study did not involve wild animals.
Reporting on sex	Sex and gender were not considered in the study design.
Field-collected samples	The study did not involve samples collected from the field.
Ethics oversight	The animal studies were reviewed by the Royal Veterinary College Animal and Ethical Review Board, and carried out in accordance with ethical standards (European Commission 2010, the Animal Welfare Act 2006 and the Welfare for Farm Animals (England) Regulations 2007).

Note that full information on the approval of the study protocol must also be provided in the manuscript.
